# Reduction of Pathological Skin-Picking Via Expressive Writing: A Randomized Controlled Trial

**DOI:** 10.32872/cpe.11215

**Published:** 2023-06-29

**Authors:** Carina Schlintl, Anne Schienle

**Affiliations:** 1 Clinical Psychology, University of Graz, BioTechMed, Graz, Austria; Philipps-University of Marburg, Marburg, Germany

**Keywords:** skin-picking, expressive writing, app-assisted approach, tension, relief

## Abstract

**Background:**

Expressive writing (EW: a personal form of writing about emotional distress, without regard to writing conventions) can improve physical and mental health. The present study investigated whether EW can reduce pathological skin-picking. In addition, the effects of two modalities of writing were contrasted with each other: computer vs. paper/pencil.

**Method:**

A total of 132 females with self-reported pathological skin-picking participated in a two-week intervention. They either carried out six EW sessions or wrote about six abstract paintings (control condition), using either paper/pencil or a computer. Before and after each session, participants rated their affective state and the urge to pick their skin via a smartphone application. Questionnaires for assessing skin-picking severity were completed before and after the two-week intervention.

**Results:**

The urge for skin-picking decreased directly after a writing session. The reduction was more pronounced in participants of the EW group, who also experienced reduced tension and increased feelings of relief at the end of a writing session. EW also reduced the severity of focused skin-picking after the two-week intervention. The writing modality had no differential effect on skin-picking symptoms.

**Conclusions:**

This study identified beneficial effects of EW on pathological skin-picking. A future study could investigate EW as a potential tool in the context of (online) psychotherapy for skin-picking disorder.

## Highlights

- Expressive writing (EW) reduces pathological skin-picking.
- EW reduces the urge for skin-picking.
- EW increases feelings of relief.
- The beneficial effects of EW are associated with trait anxiety.

Skin-picking is a common behavior in the general population. While occasional manipulation of the skin in the form of picking at scabs, bumps, or the cuticles around fingernails can be considered normal and generally as not having any negative consequences, more frequent and intense skin-picking can lead to somatic problems (skin lesions, infections, scars) and impaired socio-emotional functioning. In this case, excessive skin-picking has developed into a mental disorder, labeled as skin-picking disorder (SPD; American Psychiatric Association, 2013).

Research suggests that (benign as well as pathological) skin-picking often occurs in reaction to the experiencing of negative affective states (e.g., anger, anxiety). It usually provides short-term relief of tension and elicits positive feelings (Bohne et al., 2002). Indeed, many people who pick their skin report that they find it soothing, satisfying, and/or rewarding (Gallinat et al., 2021; Schienle & Wabnegger, 2020). Thus, skin-picking can be seen to serve emotion regulation, which can be functional (as in occasional skin-picking), or dysfunctional (as in SPD).

Several studies have shown associations between excessive skin-picking and difficulties in emotion regulation (Prochwicz et al., 2018; Schienle et al., 2018; Snorrason et al., 2010). For example, Snorrason et al. (2010) demonstrated that difficulties in emotion regulation (e.g., difficulties engaging in goal-directed behavior under distress), as well as increased emotional reactivity, predicted pathological skin-picking. A study by Schienle et al. (2018) also found strong associations between excessive skin-picking and emotion dysregulation. More specifically, the severity of focused skin-picking (i.e., skin-picking performed with full awareness, in contrast to automatic skin-picking) was predicted by difficulties in controlling impulsive behaviors, self-disgust (the tendency to feel disgusted by one's behavior), and disgust proneness (the tendency to experience disgust towards potential transmitters of disease). Further, Prochwicz et al. (2018) investigated a non-clinical sample (university students) and also found an association between a strategy for emotion regulation and skin-picking severity. It was shown in that study that those who used cognitive reappraisal more often (i.e., re-evaluation of emotion-eliciting situations/ cognitive distancing) reported a lower skin-picking severity.

The studies mentioned above suggest that excessive skin-picking might be used as an alternative strategy for controlling one’s negative emotions when other effective strategies are not at hand. Along this line of reasoning, the emotion regulation model of SPD (e.g., Snorrason et al., 2010) holds that skin-picking is an emotion regulation strategy used by people who have difficulties in applying more adaptive strategies. Based on these findings, it would appear important to offer alternative methods for emotion regulation to those who pick their skin excessively. One possible approach is expressive writing (EW).

EW can be described as personal and emotional writing without regard to form or writing conventions (e.g., spelling, punctuation, grammar). EW was first introduced by Pennebaker and Beall (1986) who asked students to write about their thoughts and feelings associated with a stressful/traumatic or neutral event. The protocol in that investigation included four writing sessions, each lasting 15 minutes. It was found that EW fostered favorable physical and mental health-related outcomes: a reduction of visits to the university health center during a 6-month follow-up period and improved well-being. Further, two meta-analyses support the notion that EW about upsetting experiences produces improvements in mood as well as in indicators of quality of life (Pavlacic et al., 2019; Reinhold et al., 2018).

The mechanisms underlying the positive effects of EW are still under investigation. Pennebaker et al. (1990) have suggested that the process of EW can help one to better understand a distressing event that has taken place (gaining insight), and further, that EW can promote better problem-solving. EW has also been suggested to support disinhibition (catharsis), self-regulation, social integration, and acceptance of the negative experience (Frattaroli, 2006; Pavlacic et al., 2019). Finally, other authors have emphasized the role of exposure in EW (Frattaroli, 2006). Participants subject to EW interventions repeatedly confront themselves with thoughts and feelings regarding an upsetting event. Similarities can be drawn between this approach and exposure (or flooding) therapy, which promotes habituation, extinction, and cognitive restructuring. Based on meta-analytical findings, Frattaroli (2006) concluded that exposure theory has received the most empirical support for explaining EW effects.

In the case of excessive skin-picking, it is very likely that EW possesses an additional positive component: The mechanical requirements of writing (either by hand or by computer) make skin-picking difficult to perform at the same time. Thus, EW incorporates a form of ‘stimulus control’ (by reducing the opportunity to perform skin-picking), which has been identified as a successful psychological treatment strategy for skin-picking disorder (Snorrason et al., 2017). Further, the process of writing – holding the pen and performing up and down movements – is somewhat similar to the physical movements involved in skin-picking. Along these lines, patients with SPD have reported that drawing (e.g., pencil sketches) can be a replacement behavior for skin-picking (Atkin, 2017). Thus, it is assumed that the process of writing in EW, particularly in the paper/pencil form, may contribute to its effectiveness in reducing skin-picking.

The present study investigated whether a two-week intervention with EW (including six writing sessions) could reduce pathological skin-picking. Short-term effects of EW (e.g., changes in the urge to pick one’s skin directly after a writing session), as well as mid-term effects (e.g., changes in self-reported skin-picking severity), were assessed. Further, the effects of two modalities of writing on the urge for skin-picking were contrasted with each other: computer vs. paper/pencil. The following hypothesis had been preregistered: Expressive writing (particularly paper/pencil writing) reduces skin-picking behavior. In addition, an exploratory regression analysis was carried out to identify variables (e.g., number of completed writing sessions, trait anxiety) that were associated with the effectiveness of expressive writing (in terms of reduction in the urge for skin-picking).

## Method

### Participants

Participants with self-reported pathological skin-picking were invited to participate in a study on the effects of different writing interventions (this was carried out via postings on social media, and self-help groups for skin-picking disorder). The invitation included a link to an online survey that checked that participants met inclusion/exclusion criteria. Inclusion criteria were female sex, because of a higher prevalence of skin-picking behavior in the female population (APA, 2013), and scores ≥ 7 on the Skin Picking Scale-Revised (SPS_R, Gallinat et al., 2016). Exclusion criteria included an existing diagnosis of a psychotic disorder, substance dependence, posttraumatic stress disorder, or depression with severe symptoms. Furthermore, participants who reported skin diseases were excluded. A total of 308 participants were eligible; of them, 158 could be contacted and agreed to participate in the study. Twenty-six participants (16%) dropped out of the study during the intervention. Data from 132 participants were included in the analyses (see Supplementary Figure 1: CONSORT flow diagram). 34% of the females participated in self-help groups during the course of the study.

The participants were randomly allocated to one of four groups: (a) Expressive Writing (paper/pencil), (b) Expressive Writing (computer), (c) Picture description (paper/pencil), (d) Picture description (computer). The four groups did not differ in the number of participants, mean age, years of education, and reported symptom severity of skin-picking as assessed by the Skin Picking Scale (SPS_R; Gallinat et al., 2016) and the Milwaukee Inventory for the Dimensions of Adult Skin-picking (MIDAS; Walther et al., 2009; *M* = 22.36, *SD* = 4.56). Moreover, participants did not differ in trait anxiety and trait depression according to the State-Trait Anxiety and Depression Inventory (STADI; Laux et al., 2013). For group characteristics see Table 1.

**Table 1:** Group Characteristics (Means, Standard Deviations, F/Chi-Square Statistics)

Characteristic	Expressive Writing (paper/pencil)	Expressive Writing (computer)	Picture Description (paper/pencil)	Picture Description (computer)	Statistics
	***M* (*SD*)**	***M* (*SD*)**	***M* (*SD*)**	***M* (*SD*)**	
Mean age (years)	28.21 (8.13)	27.71 (10.69)	30.29 (11.80)	27.50 (6.98)	*F*(3,128) = .61, *p* = .608, $\eta_{p}^{2}$ = .014
Years of education	14.09 (2.14)	13.68 (2.17)	13.68 (2.26)	14.03 (2.16)	*F*(3,128) = .34, *p* = .796, $\eta_{p}^{2}$ = .008
SPS_R	14.58 (4.15)	14.58 (3.78)	14.11 (4.13)	14.47 (4.04)	*F*(3,128) = .11, *p* = .954, $\eta_{p}^{2}$ = .003
MIDAS (focused)	22.88 (4.97)	22.55 (3.84)	21.50 (5.28)	22.70 (3.81)	*F*(3,128) = .661, *p* = .578, $\eta_{p}^{2}$ = .015
STADI_depression	20.70 (5.75)	21.45 (6.07)	21.58 (6.04)	22.00 (5.87)	*F*(3,128) = .268, *p* = .849, $\eta_{p}^{2}$ = .006
STADI_anxiety	23.97 (5.55)	23.55 (5.41)	23.74 (6.32)	25.20 (5.19)	*F*(3,128) = .530, *p* = .662, $\eta_{p}^{2}$ = .012
	***N***	***N***	***N***	***N***	
Number of participants	33	31	38	30	χ⁠^2^(3) = 1.15, *p* = .765
Dropout rate	9	3	8	6	χ⁠^2^(3) = 2.23, *p* = .527

*Note.* SPS_R = skin picking scale revised; MIDAS (focused) = subscale focused picking of the Milwaukee Inventory for the Dimensions of Adult skin picking; STADI_depression = subscale trait depression of the State Trait Anxiety and Depression Inventory; STADI_anxiety = subscale trait anxiety of the State Trait Anxiety and Depression Inventory.

All participants provided written informed consent before participating. This study was preregistered on the German Register for Clinical Studies (DRKS00029224; 2022/06/07) and approved by the ethics committee of the University (GZ. 39/79/63 ex 2021/22).

### Questionnaires

Before and after the two-week intervention participants filled out the following questionnaires via online surveys:

- German version of the Skin Picking Scale-Revised (Gallinat et al., 2016), which assesses symptom severity and impairment due to skin-picking during the last week. The eight items (e.g., How strong was your urge to pick your skin?) are answered on 5-point scales (0 = no urge; 4 = very strong urge). An overall score (total SPS_R; Cronbach’s alpha = .81) was computed that reflects the severity of skin-picking. A score of 7 represents the clinical cut-off (Gallinat et al., 2016).
- The Milwaukee Inventory for the Dimensions of Adult Skin-picking (MIDAS; Walther et al., 2009) is a self-report questionnaire with two subscales: automatic skin-picking (Cronbach’s α = 0.62; e.g., I don't notice that I have picked my skin until after it's happened.) and focused skin-picking (Cronbach’s α = 0.75; e.g. I experience an extreme urge to pick before I pick). The six items of each subscale are judged on 5-point Likert scales (1 = not at all; 5 = very much). Due to the low Cronbach’s α of the automatic skin-picking subscale, no further analyses were performed with this subscale.
- The trait version of the State-Trait Anxiety and Depression Inventory (STADI; Laux et al., 2013) has two subscales: Depression (α = .913) and Anxiety (α = .866), with ten items each (e.g., depression: “I am sad”; anxiety: “I worry that something might happen”) that are scored on a four-point Likert scale ranging from 1 (not at all) to 4 (very much).

### App-Assisted Interventions

All participants of the four intervention groups were asked to set aside at least 10 minutes for each writing session in a quiet place without disturbance. In total, six writing sessions had to be completed within a two-week period (with a maximum of one writing session per day). The participants had the option to write more than six times during the two weeks if they felt to do so. Before and after each writing session, the participants rated their affective state (pleasantness, tension, relief, urge to pick the skin) via a smartphone app on 100-point Likert scales (0 = I do not feel good, tense, relieved, no urge to pick my skin; 100 = I feel good, tense, relieved, a strong urge to pick my skin). The rating interval (pre vs. post-writing) was set to 10 minutes (it was not possible to provide the app ratings earlier).

The group-specific instructions for the writing sessions were as follows:

- *Expressive writing:* Expressive writing is an intervention in which people spend a few minutes writing about specific, personally relevant topics over several days. Let your thoughts and feelings wander freely while writing. Expressive writing has been studied since the 1980s and offers a beneficial way to engage with one's emotions and manage them. Write for at least 10 minutes about a topic that is currently on your mind. Explore your thoughts and emotions openly that you perceive while writing. Spelling, syntax, or grammar are irrelevant. It is desirable to get into a flow of writing. Choose a time of the day that suits you best and find a quiet place where you will not be disturbed (e.g., put your mobile phone in flight mode).
- *Picture description:* A picture description is a visual representation translated into language. It is meant to be a reproduction of what is seen in the picture. For example, image descriptions enable visually impaired people to find access to pictorial representations such as paintings or photographs. The detailed descriptions train analytical and structural thinking, which are important skills for problem-solving and finding new solutions. Choose a time of the day that suits you best and find a quiet place where you will not be disturbed (e.g., put your mobile phone in flight mode). Describe for at least 10 minutes one of the abstract pictures that you have received from us. Write about the appearance of the image as factually and neutrally as possible, as if you were describing it to a visually impaired person.

Half of the participants were asked to use paper and pencil to complete the task, while the other half of the participants were assigned to the computer-writing groups. The written texts remained with the participants; the experimenters had no access to the texts.

### Procedure

**Figure 1:**
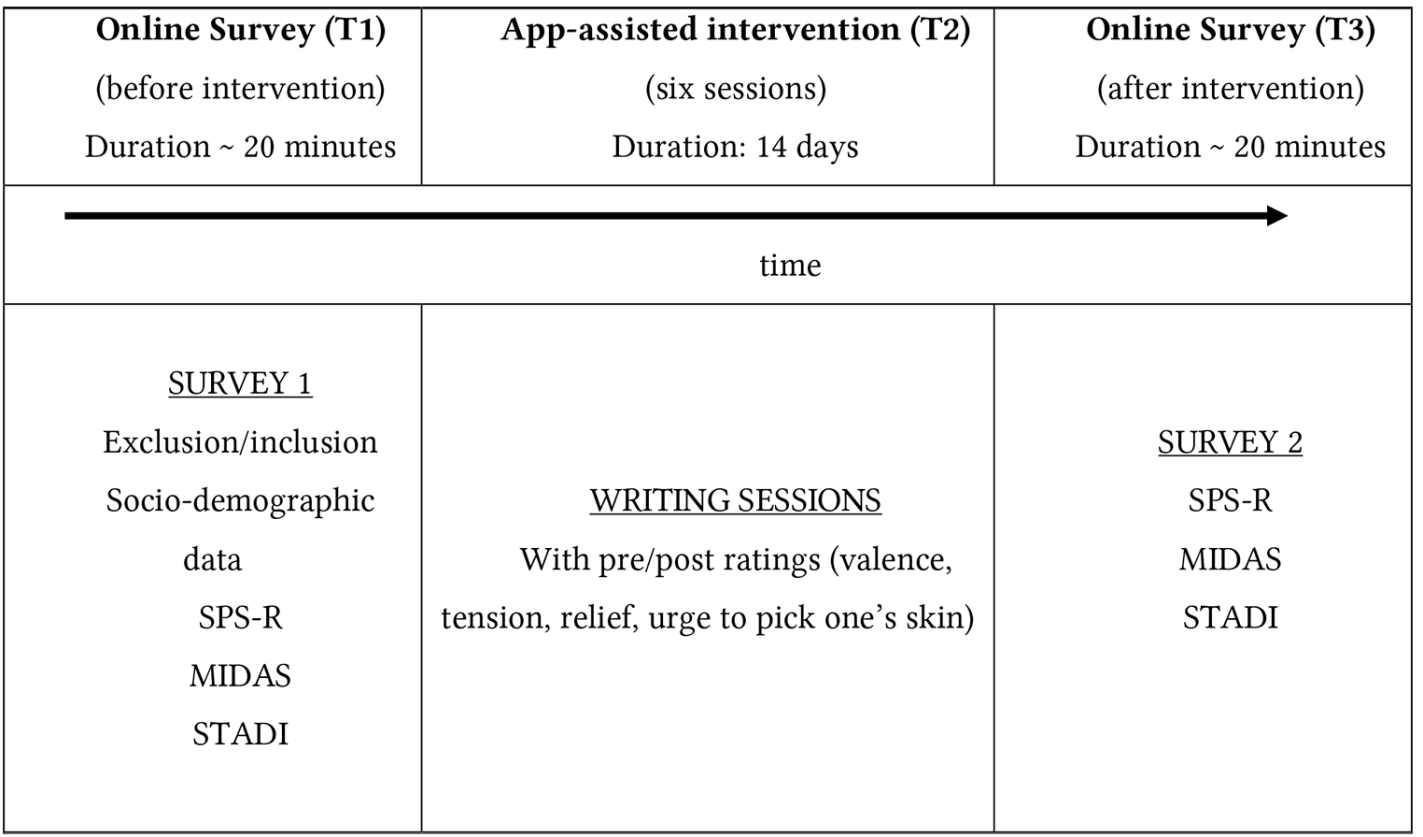
Procedure *Note.* SPS-R: skin-picking scale (revised); MIDAS (Milwaukee inventrory of the dimensions of adult skin-picking); STADI: subscales trait anxiety/ depression of the state trait anxiety and depression inventory.

After the first online survey (checking of inclusion/exclusion criteria), eligible participants were scheduled for a personal meeting where they were randomly allocated to one of four interventions: (a) Expressive Writing (paper/pencil), (b) Expressive Writing (computer), (c) Picture description (paper/pencil), (d) Picture description (computer). All participants received further information about the study, including instructions for using the smartphone app. After participants completed the two-week writing intervention, they were asked to fill out a second online survey (questionnaires). Moreover, participants were asked to count the words written in each session. We consider the number of written words as a proxy for the time spent writing. Further, we chose this measure to detect potential noncompliance (e.g., refusal to engage in writing). The procedure is depicted in Figure 1.

### Statistical Analysis

*Self-reports assessed via the smartphone app*: Mixed-model analyses of variance (ANOVAs) were conducted to compare the two INTERVENTIONS (Expressive Writing (EW) vs Picture Description (PD)) and the two WRITING MODALITIES (paper pencil (pp) vs computer (c)), before vs after a writing session (factor: TIME). This was done for the dependent measures: urge to pick one’s skin, feelings of tension, relief, and pleasantness. The ratings were averaged across the number of writing sessions during the two weeks. Moreover, word count (number of written words) was compared between the INTERVENTIONS via an ANOVA.

*Questionnaires:* Mixed-model analyses of variance (ANOVAs) were computed to compare the questionnaire scores (SPS-R; MIDAS; STADI_depression, STADI_anxiety) between INTERVENTIONS and TIME (before and after the two-week intervention).

*Exploratory regression analyses:* To identify variables (number of completed writing sessions, word count, trait anxiety, trait depression) that are associated with the effectiveness of Expressive Writing (reduction in the urge to pick one’s skin before vs. after a writing session), a multiple linear regression analysis was conducted. The model was assessed for multicollinearity (all variance inflation factors (VIFs) < 1.5; tolerance > 0.7) and residual distribution (Cook’s distance < 0.3, Durbin Watson > 1.5 and < 2.5). All analyses were conducted with SPSS version 28.

## Results

### Self-Reports Assessed Via the Smartphone App

#### Number of Completed Writing Sessions

On average, participants completed four writing sessions (range: 1-12). The number of sessions did not differ between the INTERVENTION groups, *M*_EWpp_ = 3.88, *SD* = 2.71; *M*_EWc_ = 4.55, *SD* = 2.49, *M*_PDpp_ = 3.76, *SD* = 2.39, *M*_PDc_ = 2.97, *SD* = 2.54; *F*(3,128) = 2.003, *p* = .117, $\eta_{p}^{2}$ = .045.

#### Word Count

The ANOVA that was carried out revealed that the four INTERVENTION groups differed in the number of written words per writing session, *F*(3,128) = 14.36, *p* < .001, $\eta_{p}^{2}$ = .252. Tukey post-hoc comparisons (see Supplementary Table S1) showed that the EWc group had the highest word count (*M* = 316, *SD* = 154), followed by the EWpp group (*M* = 210, *SD* = 84), the PDc group (*M* = 205, *SD* = 135), and the PDpp group (*M* = 142, *SD* = 51).

#### Urge to Pick One’s Skin

**Figure 2:**
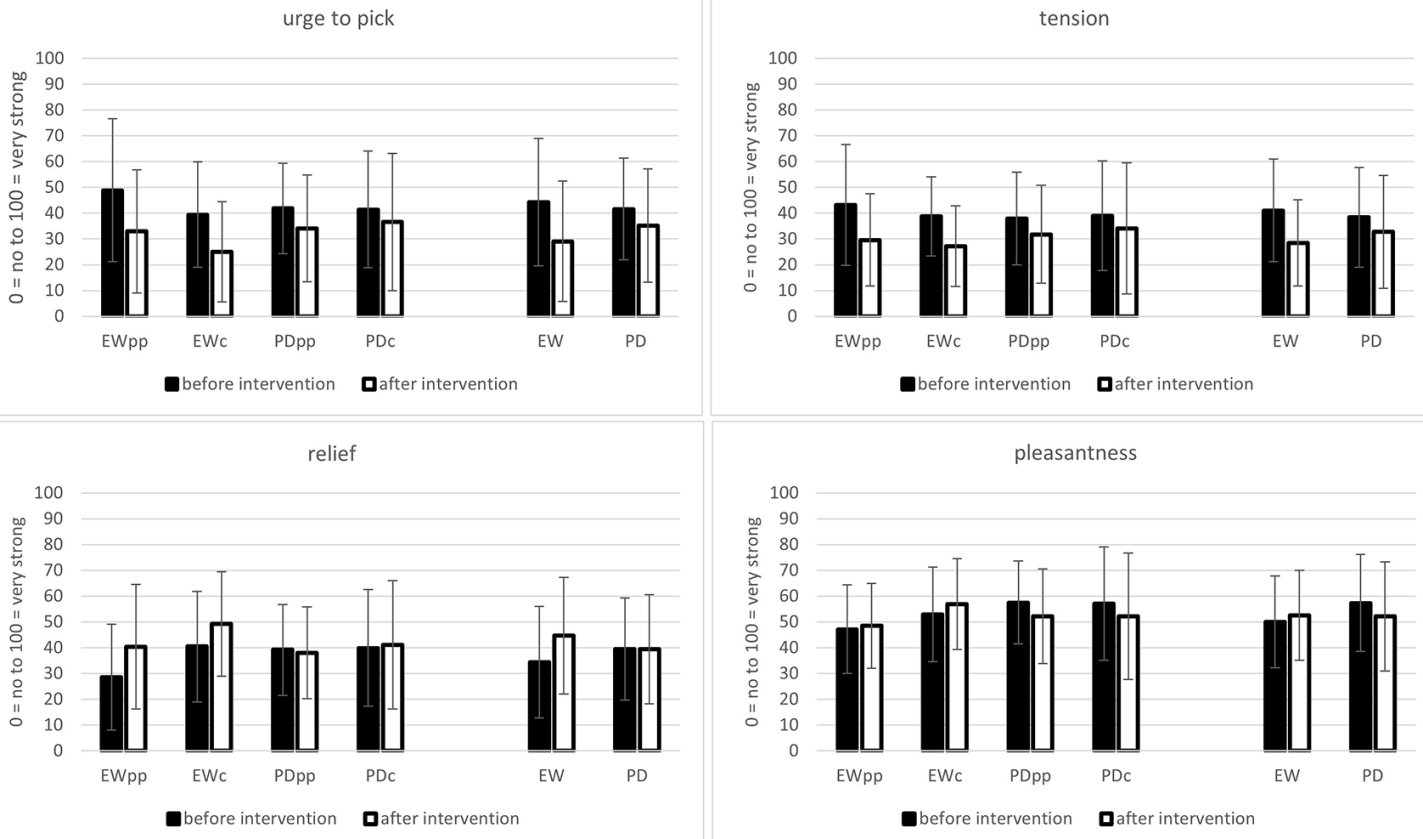
Means and Standard Deviations for the App-Data *Note.* EWpp = expressive writing paper/pencil; EWc = expressive writing computer; PDpp = picture description paper pencil; PDc = picture description computer; EW = expressive writing; PD = picture description.

The ANOVA revealed a significant main effect of TIME, *F*(1,128) = 50.64, *p* < .001, $\eta_{p}^{2}$ = .283, and a significant interaction TIME x INTERVENTION, *F*(1,128) = 8.75, *p* = .004, $\eta_{p}^{2}$ = .064. All other effects were non-significant (all *p* > .05; see Supplementary Table S2). After a session of expressive writing, participants reported a reduced urge to pick their skin compared to before the session, *t*(63) = 7.02, *p* < .001. After a session of picture description, the urge to pick was less intense compared to before the PD session, *t*(67) = 3.12, *p* = .003; Figure 2.

The reduction in the urge to pick was more pronounced in the expressive writing groups (*M*_diff_ = -15.19, *SD* = 17.30) than in the picture description groups (*M*_diff_ = -6.43, *SD* = 16.97; *t*(130) = 2.94, *p* = .004).

#### Relief

The ANOVA revealed a significant main effect of TIME, *F*(1,128) = 10.07, *p* = .002, $\eta_{p}^{2}$ = .073, and a significant interaction TIME x INTERVENTION, *F*(1,128) = 9.83, *p* = .002, $\eta_{p}^{2}$ = .071. Post hoc comparisons showed that participants felt more relieved after expressive writing than before, *t*(63) = 4.02; *p* < .001. In the picture description groups, the participants did not significantly differ in their ratings for relief before and after a writing session, *t*(67) = .04; *p* = .979; Figure 2. All other effects were non-significant (all *p* > .005; also see Supplementary Table S2).

#### Tension

The ANOVA revealed a significant main effect of TIME, *F*(1,128) = 29.95, *p* < .001, $\eta_{p}^{2}$ = .190, and a significant interaction TIME x INTERVENTION, *F*(1,128) = 4.52, *p* = .036, $\eta_{p}^{2}$ = .034. Post hoc comparisons showed that after both expressive writing, *t*(63) = 5.23; *p* < .001, and picture description, *t*(67) = 2.52; *p* = .014, participants reported reduced feelings of tension compared to before writing. The reduction of tension was more pronounced in the expressive writing groups, *M*_diff_ = -12.60, *SD* = 19.28, than in the picture description groups, *M*_diff_ = -5.61, *SD* = 18.35; *t*(130) = 2.14, *p* = .035. For means and standard deviations see Figure 2. All other effects were non-significant (all *p* > .005; also see Supplementary Table S2).

#### Pleasantness

The ANOVA revealed a significant interaction effect TIME x INTERVENTION, *F*(1,128) = 7.88, *p* = .006, $\eta_{p}^{2}$ = .058. All other effects were non-significant (all *p* > .005, see Supplementary Table S2). Post hoc comparisons revealed that participants in the picture description groups felt more pleasant than participants in the expressive writing groups before the session *t*(130) = 2.31; *p* = .023. After the session, the groups did not differ in valence ratings, *t*(130) = .12; *p* = .905. In the picture description groups, participants felt more unpleasant after the writing than before, *t*(67) = 2.56; *p* = .013. In the expressive writing group, participants did not significantly differ in their pleasantness ratings before and after the session, *t*(63) = 1.41; *p* = .165. For means and standard deviations see Figure 2.

### Questionnaire Data

#### Skin Picking Scale (Revised)

The ANOVA revealed a significant main effect of TIME, *F*(1,128) = 28.53, *p* < .001, $\eta_{p}^{2}$ = .182. After the two-week intervention, participants scored lower on the SPS-R (*M* = 12.89, *SD* = 4.72) than before (*M* = 14.42, *SD* = 4.00) independent of INTERVENTION and WRITING MODALITY. All other effects were non-significant (all *p* < .005; see Supplementary Table S3).

#### Milwaukee Inventory for the Dimensions of Adult Skin-Picking (Focused)

The ANOVA revealed a significant main effect of TIME, *F*(1,128) = 5.56, *p* = .020, $\eta_{p}^{2}$ = .042, and an interaction effect TIME x INTERVENTION, *F*(1,128) = 7.46, *p* = .007, $\eta_{p}^{2}$ = .055. Post hoc comparisons showed that participants of the expressive writing groups scored lower on the focused picking scale of the MIDAS after the intervention (*M* = 21.47, *SD* = 4.65) than before, *M* = 22.72, *SD* = 4.42; *t*(63) = 4.04, *p* < .001. In contrast, participants of the picture description groups did not differ in their scores before (*M* = 22.03, *SD* = 4.69) and after the two-week intervention, *M* = 22.10, *SD* = 4.46; *t*(67) = .20; *p* = .842. All other effects were non-significant (all *p* > .05; see Supplementary Table S3).

#### State-Trait Anxiety Depression Inventory

The ANOVA revealed no significant effects for trait anxiety and trait depression (all *p* > .05; see Supplementary Table S3).

### Regression Analysis

The regression equation for the dependent variable ‘reduction in the urge to pick one’s skin’ (before minus after a session of EW) with the predictors number of writing sessions, word count, depression, and anxiety, was significant, *R*^2^ = .17; *F*(4,63) = 2.98, *p* = .026. Trait Anxiety was a significant positive predictor. Participants with a higher level of trait anxiety showed a greater reduction in the urge to pick their skin due to expressive writing (for statistics see Table 2).

**Table 2:** Results of the Multiple Linear Regression Analysis for the Association Between “Reduction in the Urge to Pick” (Before Minus After a Writing Session) and “Number of Writing Sessions,” “Wordcount”, “STADI_Anxiety” and “STADI_Depression”

Variable	*B*	*SE B*	β	*t*	*p*	95.0% CI B		*r*	*sr*
						*LL*	*UL*		
(Constant)	-13.973	10.913		-1.280	.205	-35.810	7.864		
wordcount	.005	.016	.036	.303	.763	-.026	.036	.014	.039
frequency	-.751	.800	-.113	-.939	.352	-2.352	.850	-.106	-.121
STADI_anxiety	1.091	.461	.344	2.368	.021	.169	2.014	.390	.295
STADI_depression	.244	.431	.083	.567	.573	-.618	1.106	.263	.074

*Note. SE B* = standard error of B; 95% CI *B* = 95% confidence interval for *B*; *r* = bivariate correlation, and *sr =* partial correlation; wordcount = average number of written words per writing session; frequency = number of writing sessions; STADI_anxiety = subscale trait anxiety of the state trait anxiety and depression inventory; STADI_depression = subscale depression of the state trait anxiety and depression inventory.

## Discussion

This study investigated the effects of expressive writing (using an app-assisted approach) on excessive skin-picking behavior. Each participant was asked to complete six writing sessions over two weeks that either focused on emotional experiences with personal relevance (expressive writing), or the description of abstract paintings (control condition).

The main findings of this study were that expressive writing (EW) produced positive short-term and mid-term effects on skin-picking behavior. Directly after a writing session, the two EW groups (computer, paper/pencil) reported a reduced urge to pick their skin. Interestingly, the control groups also expressed less of an urge to manipulate their skin after describing a painting. This latter finding implies the positive effects of distraction on skin-picking behavior. This is in line with clinical recommendations which suggest, for example, distracting one’s hands with stress balls, fidgets, or tangle toys to reduce skin-picking (e.g., Snorrason, Goetz, & Lee, 2017). Similarly, cognitive-behavioral therapy for skin-picking disorder typically includes stimulus control techniques as well as habit reversal training: This involves those affected being taught to engage in harmless motor behaviors (like clenching one’s fists), which in turn prevent skin-picking (e.g., Snorrason, Goetz, & Lee 2017).

Importantly, the effects of EW on skin-picking go beyond distraction and motor control. In the present study, EW was associated with a more pronounced reduction in the urge to pick one’s skin than picture description (a reduction of -15 vs. -6 points on a scale ranging from 0 to 100). Moreover, only EW was associated with the reduction of focused skin-picking as indexed by the MIDAS. Whereas the control groups showed no change, the EW groups showed an average reduction of one point in their MIDAS scores. Thus, EW and picture description exhibited differential effects on skin-picking symptoms (with small to moderate effect sizes).

EW also demonstrated immediate effects on participants’ affective states. Directly after a writing session, participants in the EW groups reported a greater reduction of tension than those in the control groups. In addition to this, those in the EW groups also experienced increased feelings of relief (this positive emotion occurs as a response to a threat that has abated or disappeared). Previous findings have suggested that EW exerts its effects through habituation, and/or through the (re)structuring of anxious feelings (Sabo-Mordechay et al., 2019; Pennebaker & Chung, 2011; Perry & Ward-Smith, 2018). In this sense, the findings of the present study imply that EW may have assisted participants in reducing their emotional distress, which in turn reduced the need for skin-picking (i.e., the emotional distress may have no longer been pronounced enough to trigger skin-picking). This interpretation is also in line with exposure theory: When patients repeatedly confront themselves with negative feelings, this repetition and exposure can eventually lead to extinction of those feelings and associated thoughts (see Frattaroli, 2006).

An exploratory analysis was carried out which attempted to identify variables associated with the effectiveness of EW. This regression analysis showed that the number of writing sessions completed and the number of words written during a session did not contribute significantly to the positive effects of EW. In the present study, participants completed on average four writing sessions; this was below the six sessions they were originally instructed to carry out. Nonetheless, this amount of writing was sufficient to reduce skin-picking behavior. This finding is also in line with recommendations based on a meta-analysis by Frattaroli (2006) who investigated optimal conditions for EW effects; these conditions included completing a minimum of only three writing sessions. Thus, the average of four writing sessions carried out in the current study can be seen as sufficient to produce positive results.

A further finding of the current study was that there was a general trend toward more words being written on the computer compared to handwriting. This appears to reflect different writing speeds for each modality. An unexpected finding was that the writing modality had no differential effect on the reduction of skin-picking symptoms. We had assumed that the process of writing (performing up and down movements) would be similar to the physical movements involved in skin-picking, and could therefore be an efficient replacement behavior. The null findings of the current study, however, are in line with results reported in the meta-analysis by Frattaroli (2006). In that study, it was concluded that the mode of disclosure did not moderate EW outcomes; studies using handwritten disclosure did not produce larger effects than studies using typed disclosure.

The present investigation also showed that high levels of reported trait anxiety were associated with more positive effects of EW (in terms of a greater reduction in the urge to pick one’s skin). Anxiety has been shown to be a typical elicitor of skin-picking episodes (e.g., Yeo & Lee, 2017). Further, patients with skin-picking disorder report elevated trait anxiety and show elevated rates of comorbid anxiety disorders (Schienle et al., 2022). Other studies have demonstrated that EW is effective at reducing anxiety and associated problems (e.g., test anxiety; see Park et al., 2014; Robertson et al., 2021; Shen et al., 2018). For example, Park et al. (2014) showed that highly math-anxious individuals performed significantly worse on a math test than individuals with low anxiety. Notably, a subsequent EW intervention significantly reduced the group difference in test scores. The authors of that study proposed that the EW might have enabled participants to more effectively identify and differentiate their emotional experience, which may have led to the use of better emotion regulation strategies. Further, the use of specific words in the EW task related to anxiety, cause, and insight, was positively related to math performance (also see Shen et al., 2018). Thus, confrontation with anxious feelings, as well as cognitive restructuring, appear to be important components involved in the positive effects of EW on anxiety and related problems; both components are elements of exposure therapy, which is a highly effective method for reducing symptoms of anxiety and other negative emotions (e.g., Hollon & Beck, 1994; Margraf & Schneider, 1990; Ruhmland & Margraf, 2001). In the current study, while trait anxiety was not found to be reduced on average after the EW intervention, trait anxiety was however identified as a moderator for the effects of EW on the urge to perform skin-picking (i.e., participants high in trait anxiety were found to benefit more from EW). Considering this, in future EW studies that focus on excessive skin-picking, text analyses could be implemented to further elucidate anxiety-associated mechanisms of EW in the context of this dysfunctional behavior. Further, additional trait variables associated with affective processing in the context of pathological skin-picking (e.g., disgust propensity, difficulties in emotion regulation) should be investigated (Schienle et al., 2018).

It is important to mention the potential limitations of the present study. First, we only studied females. Therefore, the results cannot be generalized to males or other groups. Second, some of the participants took part in self-help groups during the study; this could have biased results. However, none of the participants received any other form of psychological treatment during the course of the study. Third, observed changes in skin-picking behavior were based on the self-reports of participants. In future studies, objective measures could be introduced (e.g., photos of affected skin before and after the EW intervention). Finally, participants received a brief intervention lasting only two weeks. The implementation of EW as an additional component in a (longer-lasting) psychotherapy would very likely enhance its effectiveness. Further, this type of psychotherapy would not have to be based on conventional face-to-face interactions but could be provided via online counseling. The present study underlines how technologies such as app-assisted interventions can be used to promote beneficial effects for reducing psychological symptoms, in this case, pathological skin-picking. Such e-therapy approaches might also enhance the effectiveness of EW interventions, since larger effects of EW have been obtained when participants have disclosed at home vs. in other (non-private) settings (Frattaroli, 2006).

### Conclusion

This study revealed positive immediate effects of EW on skin-picking, including a reduced urge for skin-picking and increased feelings of relief. Mid-term effects of EW on skin-picking were also found, relating to a reduction in focused skin-picking (according to self-reports). The beneficial effects of EW were independent of the writing modality (paper/pencil vs. computer) and were also found to be associated with trait anxiety.

## Supplementary Materials

The Supplementary Materials contain the following items (for access see Index of Supplementary Materials below):

1. The pre-registration protocol for the study.
2. Follow-up tests (Tukey post-hoc comparisons) for the analysis of variance (ANOVA) that compared the four interventions (Expressive Writing: paper/pencil; Expressive Writing: computer; Picture Description: paper/pencil; Picture description: computer) concerning word count (number of written words during a session) are provided in the Supplementary Table S1.
3. *F*-statistics (*F*, *df*, *p*, part η2) for the mixed-model analyses of variance (ANOVAs) to compare the two INTERVENTIONS (Expressive Writing (EW) vs Picture Description (PD)) and the two WRITING MODALITIES (paper pencil (pp) vs computer (c)), before vs after a writing session (factor: TIME) concerning the app ratings (urge to pick one’s skin, feelings of tension, relief, and pleasantness) are provided in Supplementary Table S2.
4. *F*-statistics (*F*, *df*, *p*, part η2) for the mixed-model analyses of variance (ANOVAs) to compare the questionnaire scores (SPS-R; MIDAS; STADI_depression, STADI_anxiety) between INTERVENTIONS and TIME (before and after the two-week intervention) are provided in Supplementary Table S3.
5. Supplementary Figure S1 depicts the CONSORT flow diagram.

SchlintlC.
SchienleA.
 (2022). Effect of expressive writing on emotions and thoughts in dermatillomania
[Pre-registration protocol; DRKS-ID: DRKS00029224]. PsychOpen. https://drks.de/search/en/trial/DRKS00029224


SchlintlC.
SchienleA.
 (2023). Supplementary materials to "Reduction of pathological skin-picking via expressive writing: A randomized controlled trial"
[Additional information]. PsychOpen. 10.23668/psycharchives.12906
PMC1050825237732151

## Data Availability

The raw data supporting the conclusions of this article will be made available by the authors, without undue reservation.

## References

[sp1_r1] Schlintl C. Schienle A. (2022). Effect of expressive writing on emotions and thoughts in dermatillomania [Pre-registration protocol; DRKS-ID: DRKS00029224]. PsychOpen. https://drks.de/search/en/trial/DRKS00029224

[sp1_r2] Schlintl C. Schienle A. (2023). Supplementary materials to "Reduction of pathological skin-picking via expressive writing: A randomized controlled trial" [Additional information]. PsychOpen. 10.23668/psycharchives.12906 PMC10508252 37732151

